# Effect of Alpha Hydroxy Acids as Dentin Conditioning Agents on Growth Factor Release: An In Vitro Study

**DOI:** 10.7759/cureus.69646

**Published:** 2024-09-18

**Authors:** Lakshmi Tulasi, Sihivahanan Dhanasekaran, Vijay Venkatesh

**Affiliations:** 1 Conservative Dentistry and Endodontics, Sri Ramaswamy Memorial (SRM) Kattankulathur Dental College, Chennai, IND

**Keywords:** dentin conditioning, edta, growth factors, regeneration, tgf- β1

## Abstract

Aim: The study aimed to evaluate the effects of alpha hydroxy acids and chelating agents on dentin conditioning for the release of growth factors.

Methods: The agents used for dentin conditioning included 17% ethylenediaminetetraacetic acid (EDTA), 10% glycolic acid (GA), 10% citric acid (CA), and 5% maleic acid (MA). Forty horizontally sectioned (SV1) human dentine slices were conditioned for 5 and 10 minutes so that the growth factor liberation reached quantifiable levels. Transforming growth factor beta-1 (TGF-β1) release and surface exposure were quantified by enzyme-linked immunosorbent assay (ELISA). Growth factor measurement required immediately removing the solutions from each of the 48-well plates (with consistent dentine surface area and weight) and freezing at -20°C so that ELISA measured the growth factors.

Results: After 5-min conditioning of dentine slices, CA was the most effective agent for growth factor release into the aqueous environment as measured by ELISA (post hoc Tukey's test p<0.05). Furthermore, dentine slices subjected to GA treatment for the same duration of time showed noticeably lower surface levels of TGF-β1 in comparison to the other agents employed.

Conclusions: Based on the findings of this in vitro study, a desirable biological growth factor-mediated effect may be gained when conditioning dentin with milder acidic or chelating agents such as CA, MA, and EDTA.

## Introduction

One potential biological strategy in dentistry is regenerative endodontic treatment, designed to promote root formation and regenerate or generate a fully functional pulp dentin complex [1]. Stem cells, growth factors, and scaffolds are the three elements of the tissue engineering triad necessary for pulp-dentin complex regeneration [2-4].

During odontogenesis, odontoblasts primarily produce a variety of growth factors that have strong biological effects and are found in the dentin matrix [3-6]. The recruitment, displacement, proliferation, and differentiation of dental stem cells are all influenced by growth factors. These growth factors have been demonstrated to be secreted upon dentin damage or during regenerative procedures. Following treatment, it is critical to assess if the entire root canal space is releasing growth factors by the dentin matrix with little equipment and ample irrigation [1-5,7,8]. Various factors, including the type and concentration of intracanal medicaments and irrigant solutions, have been researched and studied as stem cell survival and activity modulators [5,9-11]. Ferracane et al. establish that the segregated bioactive molecules can be released from the dentine matrix by a variety of compounds corresponding to the clinically used agents, including chelating and acidic dentine conditioners as well as etchants, alkaline Ca(OH)2, in addition to MTA [12].

In addition to exposing collagen fibrils and releasing bioactive molecules from the dentine matrix, acidic and chelating agents can also encourage localized demineralization. TGF-β1 release was shown to be altered by bacterial biofilms, according to the study by Ballal et al. TGF-b1 growth factor release from radicular dentin was shown to be much more responsive to MA 7% than to 17% ethylenediaminetetraacetic acid (EDTA) [13].

After conditioning human dentine slices for five and 10 minutes, Sadaghiani et al. discovered that conditioning dentine with gentler acidic or chelating compounds, including citric acid (CA) and EDTA, may result in a desired biological growth factor-mediated effect [14].

In a study conducted by Atesci et al., the highest transforming growth factor beta 1 release was observed after CA treatment followed by phosphoric acid when compared with EDTA. Regenerative endodontics aims to bring the tooth pulp-dentin complex back to health and functionality. The dental pulp, which is the only fully developed and functionally vascularized tissue found in human teeth, keeps the dentin's homeostasis intact. As a result of modern root canal therapy, teeth that have undergone endodontic treatment are vulnerable to fractures and reinfections [7].

Recent work demonstrating the regeneration of dental pulp-dentin-like tissues by cell homing regulated by growth factor administration, without the necessity for cell transplantation, offers a tangible step towards clinical application. Growth factors govern the regeneration of dental pulp and dentin through the regulation of either native or imported cells [3].

A class of chemical compounds, alpha hydroxy acids (AHA), consists of a carboxylic group with an adjacent hydroxyl group which includes CA, maleic acid (MA), glycolic acid (GA), tartaric acid, and mandelic acid. These acids are individually used in dentistry as irrigants, acid etchants, and dentin conditioning agents in low concentrations [10]. A review of the literature shows that chelating agents used in regeneration techniques do not appear to release growth factors very often.

Thus, this study aimed to evaluate the release of TGF-β1 by conditioning dentin with MA and GA at intervals of 5 and 10 minutes in addition to CA and EDTA. Therefore, the purpose of this study was to evaluate how chelating agents and alpha hydroxy acids influenced dentin conditioning for growth factor release at intervals of five and 10 minutes. The null hypothesis states that there is no statistically significant difference among the conditioning agents for release of the growth factors.

## Materials and methods

The research protocol was approved by the Institutional Ethics Committee of the SRM Medical College Hospital & Research Centre (ethics clearance number: SRMIEC-ST0123-718). With ethical approval and patient approval, 40 human third molars free of decay were obtained from the Department of Oral and Maxillofacial Surgery, SRM Kattankulathur in Chennai, India. Using a sharp scalpel, the surrounding soft tissue remnants were removed from the teeth before they were cleaned in 1% sodium azide. After that, teeth were sliced horizontally into slices between 0.8 and 1 mm thick from the occlusal surface to the cemento-enamel junction using a low-speed, water-cooled, diamond-edged rotary saw. Dentin slices were stored in deionized water until they were conditioned with dentin conditioning agents.

The total sample size (n) for the current study was estimated to be 40 by maintaining, α error as 0.05 at 95% CI, β error as 0.20, power of the test (1-β error) as 80%, and effect size (Cohen’s f statistic) as 0.5 (determined from the study by Sadaghiani et al. [14]). A total of 40 dentin slices were divided into four groups, 10 in each group were analyzed for release of growth factor after dentin conditioning. Groups were divided as follows: Experimental Group I-Maleic acid, (n=10); Experimental Group II- Citric acid (n=10); Experimental Group III- Glycolic acid (n=10) and Control Group IV- EDTA (n=10) The concentrations of dentin conditioning agents used to analyze the growth factor release are summarized in Table 1.

**Table 1:** Concentration of dentin conditioning agents EDTA: Ethylenediaminetetraacetic acid

Conditioning Agent	Concentration (%)
Maleic Acid	5
Citric Acid	10
Glycolic Acid	10
EDTA	17

The samples were subjected to ELISA testing for growth factors. In order to test the growth factors using ELISA, the dentin slices were immersed in the dentine conditioning solutions for 5 and 10 minutes in a 48-well plate with uniform dentin surface area and weight. The slices were removed from the solutions and were frozen at -20°C. TGF- β1 levels in the solutions were measured using a commercially available ELISA development kit. Avidin horseradish peroxidase, biotinylated detection antibody, and capture antibody were all included in the kit. Assay was performed in line with the guidelines provided by the manufacturer.

Statistical analyses

Data regarding TGF-b concentration (pg/mL) at 5 and 10 mins in experimental and control groups were entered into Microsoft Excel and analyzed using IBM SPSS Statistics for Windows, Version 20 (Released 2011; IBM Corp., Armonk, New York, United States). Following the assessment of ELISA, tables were derived. A non-parametric one-way ANOVA with p<0.05 and post hoc Tukey's test and t-test were used to compare groups at every point using the original data and an intragroup comparison of TGF-β concentration between experimental and control groups.

## Results

ELISA detected the release of TGF- β1 into the conditioning solution with all agents after 5 and 10 minutes of immersion. CA demonstrated the highest release of growth factor when the immersion period was increased to 10 minutes, followed by EDTA and MA.

Table 2 depicts the intergroup comparison of TGF- β1 concentration between EDTA and other experimental acids such as CA, GA, and MA using the ANOVA test with p<0.05. No significant difference was shown between the acids when conditioned for 5 minutes. 

**Table 2:** TGF-β1 concentration (pg/mL) for the experimental and control groups at 5 minutes NS: Not statistically significant; n: number of samples; F value: one-way ANOVA test; EDTA: ethylenediaminetetraacetic acid

TGF- β1 Concentration (pg/mL) at 5 minutes	n	Mean + SD	95% Confidence Interval for Mean		F value	p-value
			Lower Bound	Upper Bound		
Experimental Group I (Maleic acid)	5	116.147 ± 59.035	42.844	189.449	2.662	0.083 (NS)
Experimental Group II (Citric acid)	5	91.093 ± 61.094	15.235	166.952		
Experimental Group III (Glycolic acid)	5	23.310 ± 12.440	7.863	38.757		
Control group (EDTA)	5	70.910 ± 65.180	-10.021	151.842		
Total	20	75.365 ± 60.560	47.022	103.708		

Table 3 depicts the intergroup comparison of TGF- β1 concentration between EDTA and other experimental acids such as CA, GA, and MA using the ANOVA test with p<0.05. A significant difference of 0.008 was shown between the acids when conditioned for 10min. 

**Table 3:** TGF-β1 concentration (pg/mL) for the experimental and control groups at 10 minutes *Statistically Significant (p<0.05); n: number of samples; F value: one-way ANOVA test; EDTA: ethylenediaminetetraacetic acid

TGF- β1 concentration (pg/mL) at 10 minutes	n	Mean + SD	95% Confidence Interval for Mean		F value	p-value
			Lower Bound	Upper Bound		
Experimental Group I (Maleic acid)	5	77.506 ± 35.553	33.361	121.652	5.642	0.008*
Experimental Group II (Citric acid)	5	104.877 ± 26.313	72.205	137.549		
Experimental Group III (Glycolic acid)	5	21.759 ± 21.924	-5.463	48.982		
Control group (EDTA)	5	56.029 ± 43.947	1.461	110.597		
Total	20	65.043 ± 43.503	44.683	85.403		

Table 4 depicts post hoc Tukey's test comparison of TGF-β concentration between the experimental EDTA group and control groups that are the remaining acids (p<0.05) at 10-minute intervals, showing a significant difference between CA and GA of p=0.005.

**Table 4:** Post hoc Tukey’s test for multiple comparisons of TGF-β 1 concentration (pg/mL) between the experimental and control groups at 10 minutes *Mean difference is significant at the 0.05 level; n: number of samples; EDTA: ethylenediaminetetraacetic acid

Dependent Variable	(I) Groups	(J) Groups	Mean Difference (I-J)	p-value	95% Confidence Interval	
					Lower Bound	Upper Bound
TGF- β1 concentration (pg/mL) at 10 minutes	Experimental Group I (Maleic acid)	Citric acid	-27.370	.570	-87.168	32.427
		Glycolic acid	55.747	.072	-4.050	115.545
		EDTA	21.476	.736	-38.321	81.274
	Experimental Group II (Citric acid)	Maleic acid	27.370	.570	-32.427	87.168
		Glycolic acid	83.118*	.005	23.320	142.916
		EDTA	48.847	.131	-10.950	108.645
	Experimental Group III (Glycolic acid)	Maleic acid	-55.747	.072	-115.545	4.050
		Citric acid	-83.118*	.005	-142.916	-23.320
		EDTA	-34.270	.386	-94.068	25.527
	Control group (EDTA)	Maleic acid	-21.476	.736	-81.274	38.321
		Citric acid	-48.847	.131	-108.645	10.950
		Glycolic acid	34.270	.386	-25.527	94.068

Table 5 depicts the intragroup comparison of TGF-β1 concentration between the experimental and control groups with p-value <0.05, with no significant difference within the acids.

**Table 5:** Intragroup comparisons of TGF- β1 concentration (pg/mL) between the experimental and control groups NS: Not statistically significant; n: number of samples; t value: independent t-test; EDTA: ethylenediaminetetraacetic acid

TGF- β1 Concentration (pg/mL)	Time Point	n	Mean + SD	Mean Difference	95% Confidence Interval of the Difference		df	t-value	p-value
					Lower	Upper			
Experimental group I (Maleic acid)	at 5 minutes	5	116.147 ± 59.035	38.640	-32.429	109.710	8	1.254	0.245 (NS)
	at 10 minutes	5	77.506 ± 35.553						
Experimental group II (Citric acid)	at 5 minutes	5	91.093 ± 61.094	-13.783	-82.383	54.816	8	-0.463	0.655 (NS)
	at 10 minutes	5	104.877 ± 26.313						
Experimental group III (Glycolic acid)	at 5 minutes	5	23.310 ± 12.440	1.551	-24.445	27.547	8	0.138	0.894 (NS)
	at 10 minutes	5	21.759 ± 21.924						
Control group (EDTA)	at 5 minutes	5	70.910 ± 65.180	14.880	-66.189	95.951	8	0.423	0.683 (NS)
	at 10 minutes	5	56.029 ± 43.947						

## Discussion

This in vitro investigation looked at the biological effects of alpha hydroxy acids relevant to restorative dentistry and endodontics. Since TGF-β1 is a recognized growth factor crucial for causing dental stem cells to differentiate into cells with a mineralized phenotype, its exposure and release from dentin were examined. TGF is a multifunctional signaling molecule and a powerful cell-regulating polypeptide homodimer [15]. It is essential for cell proliferation, inflammation, matrix production, and apoptosis. TGF- β1, in particular, is a vital factor for controlling odontoblast differentiation and mineralization signaling; it also exerts a chemotactic impact on cells produced from dental papillae [16,17]. In regenerative endodontics, this chemotactic characteristic may aid in attracting the entry of stem cells inside the root canal, particularly in the direction of root canal surfaces [3].

The selection of these conditioning agents was influenced by the expanding field of endodontic research. For example, a neutral chelating agent, such as EDTA, is often employed in clinical smear layer removal and was used as a positive control because it is known to release higher levels of TGF-β1 [4]. GA was recently suggested as an alternative to phosphoric acid as a surface etchant for enamel and dentin [16]. MAs, a component of the glass ionomer system that has demonstrated its potential as a smear layer remover, are now being investigated as a root canal irrigant at concentrations between 7% and 10% [18-20]. CA, a recently found powerful root canal irrigant, can potentially remove smear layers better than EDTA at a 10% dosage [3,20-22].

During the five-minute dentin conditioning period, the aqueous solution containing 5% MA, 10% CA, 17% GA, and 17% EDTA had the highest concentration of the desired growth factor, measuring 116.147 ±59.035, 91.093 ±61.094, and 70.910 ±65.180, respectively.

The null hypothesis has been rejected as, at 10 minutes of conditioning, CA showed the highest significant difference with GA with 0.008 p-values at 10 minutes of dentin conditioning with mean values 104.877 ±26.313 and 21.759 ±21.924, respectively. Whereas maleic acid 5% showed a mean value of 77.506 ±35.553, EDTA showed 56.029 ±43.947 as a mean value for the release of TGF-β1 from dentin slices.

Similar to the findings of Sadaghiani et al., release of TGF- β1 at 10 minutes of duration was statistically more for CA when compared to GA [14]. CA is a weak organic acid, frequently utilized in clinical procedures as a root conditioning agent as an irrigant during root canal therapy [3,22-24], has the ability to release matrix metalloproteinases (MMPs) from dentin and has also been shown to be superior to GA and EDTA. The ability of CA to release MMPs from dentin has also been shown to be superior to EDTA [21,22]. Lower release of growth factors by GA could be accounted for by the low pH contributing to dentine demineralization and the broken-down structure of the matrix over time, leading to an increase in the number of proteins discharged from the layers beneath it [25,26].

In agreement with previously done research, this study also found that acidic environments may activate TGF-β1 and break down the latent associated peptide. TGF-1 was significantly activated after treatment with extreme pH levels (1.5) but only to a lesser extent after treatment with mild acid (pH 4.5) [27-30]. Concurrent to the study done by Ballal et al., in which 7% MA was shown to release more growth factors when compared to 17% EDTA in the presence of a biofilm, our study has shown the highest release of growth factor with 5% MA at 5minutes duration. However, there was no statistically significant difference among the other dentine conditioning agents at 5 minutes duration which could be due to the sample size variations.

The conditions that are used here are quite longer than those that are recommended for clinical use. According to Hashimoto et al., and West et al., extended use of acidic agents in clinical settings can lead to degradation of enamel and dentine and negatively impact the restoration's bond strength [11,31]. In this investigation, we did, however, use accelerated conditions, beginning at a baseline conditioning duration of five minutes, to facilitate the detection and comparison of growth factor liberation. Because of this, even though it is well known that dentine is conditioned for much shorter periods of time in a clinical context, we decided to extend the conditioning period to ten minutes. This allowed us to better understand the growth factor release kinetics over time by adhering to the procedure by Sadaghiani et al [14].

Acidic environments can cause ELISA assays to malfunction. Some irrigants appear to underestimate the release of growth factors because of changes in amino acids and the dissociation of binding proteins brought on by protonation, which might block the target protein's signal and generate competition for binding locations with ELISA antibodies. However, as ELISA was the most often utilized technique in the papers that were considered, it would still be appropriate for this evaluation if these drawbacks could be minimized. Additional buffering solution might be introduced after the irrigants are used, enabling sample collection and assessment in this buffered solution following the irrigation operation. A crucial methodological concern is the clinical applicability of the selected experimental windows. In a clinical setting, extended exposure to demineralizing agents seems unfeasible.

## Conclusions

Considering the results of this in vitro investigation, conditioning dentin for 5 or 10 minutes with milder acidic or chelating agents such as 10% CA, 5% MA, or 17% EDTA may result in the desired biological growth factor-mediated impact. 10% CA showed the highest release and 10% GA showed the least release of growth factors.
